# Physicians' communication skills with patients and legal liability in decided medical malpractice litigation cases in Japan

**DOI:** 10.1186/1471-2296-9-43

**Published:** 2008-07-25

**Authors:** Tomoko Hamasaki, Tadamichi Takehara, Akihito Hagihara

**Affiliations:** 1 Division of Community Oral Health Science, Kyushu Dental College, Kokurakita-ku, Kitakyushu 803-8580, Japan; 2 Department of Health Services Management and Policy, Kyushu University Graduate School of Medicine, Higashi-ku, Fukuoka 812-8582, Japan

## Abstract

**Background:**

In medical malpractice litigations in recent years in Japan, it is notable that the growing number of medical litigation cases includes the issue of a doctor's explanation to the patient as a pivotal point. The objective of this study was to identify factors of physicians' communication skills with patients, as related to their legal liability, and differences in doctors' communication skills with patients by the type of medical facility.

**Methods:**

Decisions of medical malpractice litigation cases between 1988 and 2005 in Japan, the pivotal issue of which was a physician's explanation, were analyzed in the study. The content of each decision was summarized using the study variables (information about the patient, doctor, manner of the doctor's explanation, and subsequent litigation), and a database comprising the content of each decision (*N *= 100) was constructed. In order to evaluate an association between doctors' communication skills with patients and the outcome of the litigation, the analysis was performed based on the outcome of litigation or the type of medical facility.

**Results:**

The ratio of acknowledged physician liability by court decision was lower in cases in which the doctor's explanation occurred before treatment or surgery (*p *= 0.013). The ratio of acknowledged physician liability by court decision was higher in cases of elective or non-urgent treatment (*p *= 0.046). The ratio of acknowledged physician liability by court decision was higher in clinics than in hospital groups (*p *= 0.036).

**Conclusion:**

These findings are beneficial for the prevention of medical disputes and improvement of patient-physician communication.

## Background

In recent years, it has become evident that doctors' explanations to patients and patients' understanding of these explanations in medical settings are associated with broader factors. The doctor's explanation and the patient's understanding of this explanation have been shown to influence patient satisfaction and adherence to medical regimen [1-3]. Along with an increase in the number of medical malpractice litigations in recent years, factors potentially responsible for the incidence of medical disputes have been explored. As of today, poor communication skills with patients by physicians are the main cause of medical disputes [4-8]. Thus, we can safely say that doctors' communication skills with patients are extremely important for the prevention of medical disputes, as well as for patient satisfaction.

When medical malpractice litigations in recent years in Japan are surveyed, it is notable that the growing number of medical litigations includes the issue of the doctor's explanation to his/her patient as a pivotal point. The purposes of a doctor's explanation are as follows: to obtain a patient's consent in case of invasive care, to secure a patient's right of self-determination, to explain about the factors in case of negative outcomes related to patient care, and to give medical treatment guidance [9]. With respect to the doctor's explanation, a legal problem exists about the level of the physician's explanation. Concerning this point, a ruling view on the criteria for negligent and non-negligent care in Japan is useful. Non-negligent care is regarded as medical care, the level of which is equivalent to that of standard care when the care was delivered [10]. Since a doctor's duty to explain constitutes an important aspect of standard care, the nature of a doctor's explanation is also judged as a requirement for standard care when that care was delivered. Various arguments have been forwarded concerning the distinction between negligent and non-negligent care. Given that the criterion between negligent and non-negligent care is universal, no differences should exist between large medical facilities (i.e., university hospitals and general hospitals) and small solo practice clinics [11]. Recently, however, it was ruled that the characteristics of medical facilities and regions should be considered in judging the issue of whether the care provided was negligent or non-negligent [12,13].

Since the required level of the doctor's explanation must be determined in view of the requirements for standard care, the conditions, content, range, and necessity of a doctor's explanation should vary in the future. As of today, however, we do not know the answers to the following questions: Are there differences between larger hospitals (i.e., university or general hospitals) and small clinics with respect to required doctors' communication skills with patients? If there are differences, what are they?

Information on doctors' communication skills with patients is extremely limited in Japan. As decisions of litigated medical malpractice cases provide useful information about patient-physician interactions, we have analyzed litigated medical malpractice cases in Japan. We have examined the association between doctors' communication skills with patients and legal liability, and revealed that doctors' communication skills with patients, together with how they listen and talk to patients and their families, were related to the probability of a court decision of negligent care [14]. However, variables concerning patient-physician communication were extremely limited in that study, such as who received the doctor's explanations (i.e., patient, family, or both) and the manner of the doctor's interaction with the patient or family (i.e., explaining, listening, or both). In addition, we did not examine, in that study, whether the doctor's explanation to patients varied depending upon the type of medical facility.

Therefore, in this study, in making use of the decisions of litigated medical malpractice cases in Japan where the main issue was the doctor's explanation, we identified (1) factors of doctors' communication skills with patients related to physicians' legal liability, and (2) differences in doctors' communication skills with patients by the type of medical facility (i.e., large hospitals and small clinics). The database of the present study includes factors related to patient-physician communication in great detail. Our findings may be useful for improving doctors' communication skills with patients in medical settings.

## Methods

### Data Source

One hundred decisions of medical malpractice cases were analyzed in the study. Specifically, decisions were collected of litigated medical malpractice cases reported in *HanreiJiho *and *HanreiTaimuzu *between 1990 and 2005 in which the pivotal issue was physician explanation. *HanreiJiho *and *HanreiTaimuzu *report on court decisions of litigated cases in Japan.

Under the direction of one of authors (TH), three students at Kyushu Dental College read the decisions carefully. Before reading the decisions, sessions on the structure of a decision form, variables related to physician explanation, and patient and physician factors were held to educate the students. One of the authors (TH) read all the decisions, and each student carefully read about 33 decisions. After completing the reading of the decisions, and using the study variables, the content of each decision was summarized, and a database comprising the content of each decision (*N *= 100) was constructed.

In order to verify the validity of data coding, the Kappa measures of agreement were calculated with respect to the nine variables concerning each doctor's explanation. With respect to the nine variables shown in Table 1, Kappa measures of inter-rater agreement between one of the authors (TH) and three students were calculated. We obtained the following values: 0.77, 1.0, and 1.0 for the first variable, 0.91, 0.96, and 0.87 for the second, 0.94, 0.94, and 0.87 for the third, 0.94, 0.94, and 0.87 for the third, 1.00, 1.00, and 0.74 for the fourth, 0.80, 1.00, and 0.76 for the fifth, 0.91, 1.00, and 0.76 for the sixth, 0.61, 1.00 and 1.00 for the seventh, 0.61, 1.00, and 1.00 for the eighth, and 0.82, 0.81, and 0.83 for the ninth variable. These findings imply that there was good agreement among the raters. With respect to the variables whose coding was split among raters, the raters talked about a difference on the basis of coding criteria, and coding was unified.

**Table 1:** Profile of the study variables (*n *= 100)

	**Items**	**Mean ± SD (n) or number of cases (%)**	
Patient characteristics	Age (years)		40.41 ± 19.72 (54)
	Sex	Male	35 (35.0)
		Female	65 (65.0)
	Type of disease	Treatment is elective/not urgently necessary	16 (16.0)
		Other^a^	84 (84.0)
	Severity of injury	Death	43 (43.0)
		Other^b^	57 (57.0)

Physician characteristics	Number of doctors who explained	One	73 (73.0)
		Two or more	27 (27.0)
	Department where patients were treated	Surgical system	34 (34.0)
		Other^c^	66 (66.0)
	Type of medical facility	Clinic	27 (27.0)
		Hospital	73 (73.0)

Manner of doctor's explanation	Purpose of explanation	Explanation to obtain patient's consent	80 (80.0)
		Other^d^	20 (20.0)
	Timing of doctor's explanation	Prior to treatment or surgery	71 (70.0)
		After treatment or surgery	10 (10.0)
		No explanation	19 (20.0)
	Those who received doctor's explanation	Patient only	36 (36.0)
		Patient and family/family only	45 (45.0)
		No explanation	19 (19.0)
	Manner of doctor's explanation to patient	Oral only	44 (44.0)
		Oral and other methods	18 (18.0)
		No explanation/no explanation to patient	38 (38.0)
	Manner of doctor's explanation to family	Oral only	36 (36.0)
		Oral and other methods	6 (6.0)
		No explanation/no explanation to family	58 (58.0)
	Level of doctor's explanation to patient	Relevant and specific	20 (20.0)
		Not sufficiently relevant or specific	40 (40.0)
		Unknown/no explanation/no explanation to patient	40 (40.0)
	Level of doctor's explanation to family	Relevant and specific	14 (14.0)
		Not sufficiently relevant or specific	27 (27.0)
		Unknown/no explanation/no explanation to family	59 (59.0)
	Place of doctor's explanation	Inpatient ward	55 (55.0)
		Outpatient clinic/other	45 (45.0)
	Content of doctor's explanation	Related to surgery	37 (37.0)
		Other^e^	63 (63.0)

Litigation	Mean length of litigation (years)		7.62 ± 4.41 (65)
	Legal basis of plaintiff's claim	Tort law only	30 (30.0)
		Tort law and contract law/contract law only	69 (69.0)
		Other^f^	1 (1.0)
	Introduction of medical expert witness	Yes	34 (34.0)
		No	66 (66.0)
	Issue in litigation	Presence of doctor's explanation	19 (19.0)
		Insufficient/incorrect doctor's explanation	81 (81.0)
	Acknowledgement of doctor's fault by court decision	Yes	35 (35.0)
		No	62 (62.0)
		Unknown	3 (3.0)
	Acknowledgement of physician liability by court decision	Yes	_65(65.0)_
		No	_35(35.0)_

a: "Other" includes "treatment is urgently necessary" and "other". b: "Other" includes temporary or permanent injury. c: "Other" includes internal medicine, pediatrics, obstetrics, ophthalmology, otolaryngology, dermatology, dentistry, urology, and others. d: "Other" includes explanation about medical treatment guidance, and explanation about reasons of negative outcomes. e: "Other" includes explanations about medical treatment and medical testing. f: "Other" includes Constitutional rights.

### Study Variables

#### Patient characteristics

Table 1 shows the variables concerning patients, physicians, and the manner of physician explanation. Of the variables concerning patient characteristics, "type of disease" has two subcategories: "treatment is elective/not urgently necessary" and "other." The physician's duty to make an explanation to a patient is severely judged in the field of cosmetic surgery, where treatment is elective [15]. There is a difference between cosmetic surgery and other medical treatments with respect to the criteria required in the physician's explanation to a patient. Thus, these two categories were created for "type of disease." "Severity of injury" was subdivided into the categories of "death" and "other." "Other" includes temporary or cured injury, and permanent or not cured injury.

#### Doctor characteristics

Of the variables concerning doctor characteristics, the "number of doctors who explained" was placed into "one" and "two or more" categories. It has been reported that poor patient-physician communication was predictive of medical claims among internists but not among surgeons [8]. Therefore, based on this finding, "department where patients were treated" was split into two subcategories, "surgical system" and "other." The type of medical facility was classified into "clinic" and "hospital," based on the findings in the decision. Incidentally, according to medical law in Japan, "a medical institution having hospitalization institutions with more than 20 beds" is defined as a hospital, and "a medical institution having a hospitalization institution of fewer than19 beds" is defined as a clinic (Medical Law, Article 5, Law No. 205, 1948). Although age of a physician was an important factor, however the information was not obtained from the court decisions.

#### Manner of doctor's explanation

Nine types of physician communication skills with patients were examined in the study. "Purpose of explanation" has two categories, "explanation to obtain patient's consent" and "other." As a rule of thumb, the purposes of physician explanations are (1) explanation to obtain the patient's consent, (2) explanation for medical treatment guidance, and (3) post-medical treatment explanation. Of these, explanation to obtain patient's consent is related to a patient's right of self-determination, implying that explanation to obtain a patient's consent differs from other purposes of explanation. Therefore, "purpose of explanation" is split into two subcategories, "explanation to obtain a patient's consent" and "other." "Timing of doctor's explanation" was divided into three categories: "prior to treatment or surgery," when the patient received the doctor's explanation before the treatment or surgery; "after treatment or surgery," when the patient received the doctor's explanation after the treatment or surgery; and "no explanation," when no explanation was provided. In Japanese medical settings, a family tends to play an important role when receiving physician's explanation. Therefore, "those who received the doctor's explanation" had three categories: "patient only," "patient and family/family only," and "no explanation." "Manner of doctor's explanation to patient" and "manner of doctor's explanation to family" were subdivided into three categories: "oral only," "oral and other methods," and "no explanation/no explanation to patient." Other methods include documents and pamphlets. When no mention was made of the manner of physician's explanation in the decision, the case was classified as "oral only." As for "level of the doctor's explanation to the patient" and "level of the doctor's explanation to the family," "relevant and specific" was allocated to this variable when the explanation was regarded as "relevant and specific" to the decision, and "not sufficiently relevant or specific" when the explanation was regarded as "not sufficiently relevant or specific" to the decision. In other cases, "unknown/no explanation/no explanation to patient (family)" was allocated to this variable. "Place of doctor's explanation" was classified into "inpatient ward" or "outpatient clinic/other." When treatment is closely related to a patient's life or health, it is generally recognized that a doctor needs to explain fully what is happening to a patient. Thus, "content of the doctor's explanation" is categorized into "related to surgery" or "other."

#### Variables related to litigation

Lastly, we refer to variables related to litigation. "Issue in litigation" was classified into either "presence of a doctor's explanation" or "insufficient/incorrect explanation." In medical malpractice litigation, tort law, contract law, or both, are used as the legal foundation when patients file their claims. In recent years, it has been assumed that there is little difference in the burden of proof between cases using tort law and cases using contract law as the legal foundation [16]. However, it should be noted that the burden of proof may vary with the legal basis of patients' claims. In particular, patients can expect a favorable decision when breach of contract is used as the legal basis of their claims, because the level required of a physician's explanation becomes higher in these cases than in those using tort law as the legal basis [17]. Thus, "legal basis of plaintiff's claim" was categorized into "tort law only" or "tort law and contract law/contract law only." In medical malpractice litigation, an issue related to highly professional knowledge is often included, and a medical expert witness is introduced. The introduction of a medical expert witness might affect the progress and results of litigation. Thus, "introduction of a medical expert witness" was categorized as "yes" or "no." As for "acknowledgement of doctor's fault by court decision," "yes" was allocated to this variable when a mistake in a medical maneuver, error in a physician's judgment, or both, is acknowledged in the decision, and "no" was allocated to this variable in other cases. As for "acknowledgement of physician liability by court decision," "yes" was allocated to this variable when the case was decided in favor of plaintiffs (i.e., patients and their families) at a local court, a high court, or the supreme court, and "no" was allocated in other cases.

### Statistical Analysis

To evaluate an association between a physician's communication skill with patients and the outcome of litigation, Student's *t*-test for continuous variables and the χ^2^-square test for categorical variables were used. The analysis was undertaken by the litigation outcome or by the type of medical facility. The statistical software package (SPSS for Windows ver.11) was used for the analysis.

## Results

Table 1 shows variables on the patient, doctor, manner of doctor's explanation, and litigation. The mean age of the patients was 40.4 years, and 65% of them were female. Treatment was elective or not urgently needed in 16% of the cases presented for consultation, and 84% required treatment. As for the severity of the injury, the death of the patient occurred in 43% of the cases. Two or more doctors explained things in 27% of the cases, and the surgical department was most commonly involved (34% of cases). Concerning the type of medical facility, 73% involved hospitals and 27% private clinics. Concerning the purpose of doctors' explanations, 80% constituted an explanation to obtain the patient's consent.

Regarding the timing of the explanation, 71% of the explanations were given prior to treatment or surgery, 10% of the explanations were given after the treatment or procedure, and no explanation at all was given in 19% of cases. A doctor's explanation was given to the patient only in 36% of cases, and to the patient and family, or the family only, in 45% of cases. Regarding the manner of the doctor's explanation to the patient, oral explanation only was provided in 44% of cases, while oral together with other methods were given in 18% of cases. Regarding the doctor's explanation to family, oral explanation only accounted for 36% of cases. Concerning the level of doctor's explanation to the patient, 40% of cases involved relevant and specific information, while 20% did not. Additionally, doctors gave families relevant and specific details in 14% of cases, while 27% of such cases were given information that was not so concrete. The explanation occurred in the inpatient ward in 55% of cases, and the content of the doctor's explanation was related to surgery in 37% of cases.

The issue in litigation was the presence of doctor's explanation in only 19% of cases, as compared to 81% involving insufficient or incorrect explanations by the physician. In the litigation cases, 34% involved an expert medical witness, while 66% did not. The mean length of medical malpractice litigation was 7.62 ± 4.41 years. Acknowledgement of the doctor's fault, by court decision was, was found in 35% of cases. Acknowledgement of physician liability, by court decision, was found in 65% of cases, whereas physician liability was not identified in 35% of cases. Furthermore, the number of cases in which the court acknowledged physician liability was as follows. Of 65 cases, a breach of the doctor's duty to explain was noted in 30 cases, doctor's faults such as technical errors, misjudgment, and diagnosis error were noted in three cases, and both a breach of the doctor's duty to explain and doctor's fault were noted in 32 cases (data not shown).

In Table 2, the mean or ratio of each of the study variables based on court decisions of physician liability was compared. The mean age of the patient in which the court's decision acknowledged physician liability was significantly younger (40.41 ± 19.72 years) than those in which the court's decision did not acknowledge physician liability (50.53 ± 20.62 year) (*p *= 0.029). In the type of disease treatment, the ratio of elective or not urgent treatment was significantly higher in decisions in favor of the patient versus the decision in favor of the doctor or hospital (*p *= 0.046). On the other hand, the severity of injury was assessed as the death of the patient, and the ratio of decisions in favor of the doctor or hospital was higher compared to the decision in favor of the patient (*p *= 0.036). For the type of medical facility, the decision in the patient's favor was significantly higher for clinics in comparison with the decision in favor of the doctor in a hospital (*p *= 0.036).

**Table 2:** Comparison of study variables by court decision of physician liability

**Category**	**Study variable**	**Court decision acknowledged physician liability**	**Court decision did not acknowledge physician liability**	**p-value**^a^
Patient characteristics				
	Age (mean ± SD)(years) (n)	40.41 ± 19.72 (54)	50.53 ± 20.62 (30)	0.029
	Sex: male/female	24/41	11/24	0.583
	Type of disease: treatment is elective or not urgently necessary/other	14/50	2/33	0.046
	Severity of injury: death/other	23/42	20/15	0.036

Doctor characteristics				
	Number of doctors who explained: one/two or more	50/15	23/12	0.229
	Department where patients were treated: surgical system/other	21/44	13/22	0.626
	Type of medical facility: clinic/hospital	22/43	5/30	0.036

Manner of doctor's explanation				
	Purpose of explanation: explanation to obtain patient's consent/other	50/15	30/5	0.295
	Timing of doctor's explanation: prior to treatment or surgery/other	43/10	28/0	0.013
	Those who received a doctor's explanation: patient only/patient and family or family only	27/26	9/19	0.105
	Manner of doctor's explanation to patient: oral only/oral and other methods	30/12	14/6	0.908
	Manner of a doctor's explanation to family: oral only/oral and other methods	23/3	13/3	0.658
	Level of doctor's explanation to patient: relevant and specific/not sufficiently relevant or specific	4/37	16/3	0.000
	Level of doctor's explanation to family: relevant and specific/not sufficiently relevant or specific	1/24	13/3	0.000
	Place of doctor's explanation: inpatient ward/outpatient clinic or other	32/33	23/12	0.114
	Content of doctor's explanation: related to surgery/other	25/40	12/23	0.680

Litigation				
	Issue in litigation: presence of doctor's explanation/insufficient or incorrect doctor's explanation	12/53	7/28	0.852
	Legal basis of plaintiff's claim: tort law only/tort law and contract law or contract law only	20/44	10/25	0.782
	Introduction of medical expert witness: yes/no	21/44	13/22	0.626
	Mean length of litigation (mean ± SD)(years) (n)	7.62 ± 4.41(65)	7.66 ± 3.38(35)	0.961
	Acknowledgement of doctor's fault by court decision: yes/no	35/27	0/35	0.000

^a^*t*-test or χ^2 ^test

Concerning the manner of the doctor's explanation, the ratio between the timing of the doctor's explanation prior to treatment or surgery was significantly higher in decisions in favor of the doctor or hospital's favor than in the patient's favor (*p *= 0.013). With regard to the level of the doctor's explanation to the patient and to the family, the ratio of the decision in the patient's favor to that of the physician or hospital was significantly lower when the doctor provided a relevant and specific explanation (*p *= 0.000 for both variables together). The ratios of the remaining variables concerning the manner of the doctor's explanation in the decision of the court were not statistically significant.

Finally, regarding litigation, the ratio of the acknowledgment of the doctor's fault by the courts to the decision in the patient's favor was higher compared with the decision in favor of the doctor or hospital (*p *= 0.000).

The differences in the variables based on the types of medical facility were compared in Table 3. Concerning patient characteristics, the mean age was younger in the clinic group, as compared to the hospital group (*p *= 0.042). With regard to the treatment of the diseases, the ratio of elective or not urgently required treatment was higher in the clinic group as compared to the hospital group (*p *= 0.000). Regarding the severity of injury, the hospital group had a higher death ratio than the clinic group (*p *= 0.003).

**Table 3:** Comparison of study variables by type of medical facility

**Category**	**Study variables**	**Clinic**	**Hospital**	**p-value**^a^
Patient characteristics				
	Age (mean ± SD)(years) (n)	35.90 ± 17.85 (20)	46.56 ± 2 0.76 (64)	0.042
	Sex: male/female	6/21	29/44	0.103
	Type of disease: treatment is elective or not urgently necessary/other	13/14	3/69	0.000
	Severity of injury: death/other	5/22	38/35	0.003

Doctor characteristics				
	Number of doctors who explained: one/two or more	27/0	46/27	0.000
	Department where patient was treated: surgical system/other	3/24	31/42	0.004

Manner of doctor's explanation				
	Purpose of explanation: explanation to obtain patient's consent/other	19/8	61/12	0.143
	Timing of doctor's explanation: prior to treatment or surgery/other	18/4	53/6	0.448
	Those who received doctor's explanation: patient only/patient and family or family only	19/3	17/42	0.000
	Manner of doctor's explanation to patient: oral only/oral and other methods	5/14	39/4	0.000
	Manner of doctor's explanation to family: oral only/oral and other methods	3/0	33/6	1.000
	Level of doctor's explanation to patient: relevant and specific/not sufficiently relevant or specific	6/12	14/28	1.000
	Level of doctor's explanation to family: relevant and specific/not sufficiently relevant or specific	0/3	14/24	0.539
	Place of doctor's explanation: inpatient ward/outpatient clinic or other	2/25	53/20	0.000
	Content of doctor's explanation: related to surgery/other	11/16	26/47	0.637

Litigation				
	Issue in litigation: presence of doctor's explanation/insufficient or incorrect doctor's explanation	5/22	14/59	0.940
	Legal basis of plaintiff's claim: tort law only tort law and contract law or contract law only	8/19	22/50	0.929
	Introduction of medical expert witness: yes/no	2/25	32/41	0.001
	Mean length of litigation (mean ± SD)(years) (n)	6.41 ± 5.29 (27)	8.08 ± 3.43 (26)	0.067
	Acknowledgment of doctor's fault by court decision: yes/no	11/14	24/48	0.339
	Acknowledgment of physician liability by court decision: yes/no	22/5	43/30	0.036

^a^*t*-test or χ^2 ^test

In terms of the doctors' characteristics, a group of doctors in the hospital group provided information to the patient, with or without the family, more often than the clinic group did, where a single doctor usually provided information (*p *= 0.000). In addition, the ratio of the surgery department, where patients were treated, was significantly higher in the hospital group than in the clinic group (*p *= 0.004).

For the manner of the doctor's explanation, the ratio wherein a family was included was higher in the hospital group than in the clinic group (*p *= 0.000). Regarding the manner of the doctor's explanation to the patient, compared with the hospital group, a greater ratio in the use of oral and other supplemental methods, as compared to oral only, was noted in the clinic group (*p *= 0.000). The place of the doctor's explanation tended to be the inpatient ward, which occurred more often in the hospital group than in the clinic group (*p *= 0.000).

Finally, regarding the litigation variable, the hospital group had a higher ratio with respect to involving an expert medical witness, as compared to the clinic group (*p *= 0.001). Conversely, the clinic group had a higher ratio with respect to acknowledgement of physician liability by court decision than did the hospital group (*p *= 0.036).

## Discussion

### 1. Associations between doctors' communication skills with patients and legal liability

There were notable findings with respect to the association between doctors' communication skills with patients and their legal liabilities. First, there is the factor of the timing of the doctor's explanation, a subject that has not previously been studied. We studied whether the timing and manner of doctors' explanations influenced the judgment of physician liability by court decision. Our results showed that the ratio of cases in which an explanation was given prior to treatment or surgery was high in relation to the court's decision in favor of the doctor or hospital (Table 2). This shows the importance of explaining a procedure or regimen before performing the procedure.

Second, there is the factor of the level or manner of the doctor's explanation to the patient. When explanations were not relevant and specific, as occurred in most cases, decisions were in favor of the patient (Table 2). In the UK, this has increased the demand for relevant and specific explanations [17]. In Japan, the need for an explanation in layman's term is definitely required, especially for the patient who does not have professional medical knowledge [18]. In this study, when the doctor's explanation was too general, the doctor's explanation behavior was not acknowledged by the court. Hence, a more detailed dialogue between the two parties is suggested.

Third, regarding the acknowledgement of doctor's fault by a court decision, no case that was rejected (i.e., decided to be in favor of the doctor or hospital) acknowledged physician liability (Table 2). This suggests that the court's acknowledgment of a doctor's fault, including faulty management and technical mistakes, strongly influences the acknowledgment of physician liability in a court decision. In this study, 56% of such cases accounted for a court decision in favor of the patient. However, overall medical malpractice litigation in which the decision has been made in the patient's favor is low in Japan. The mean proportion of decisions in favor of patients between 1992 and 2001 was 37.42% for medical malpractice cases and 86.07% for litigated cases in general, including medical malpractice cases [19]. This could be the reason why doctors and hospitals, as defendants, are thought to be well-off and more familiar with procedures in litigations than the plaintiffs (i.e., the patients or their families). In addition, it is extremely difficult to assign liability to the doctor, insofar as there is no admission of the physician's fault in the court decisions examined [20]. This study does not include all medical malpractice litigations in Japan, but the results suggest that part of a doctor's liability includes his or her duty to explain medical issues to the patient, even without a doctor's fault such as faulty management or a technical mistake.

Fourth, the ratio of non-life-threatening diseases in which urgent management is not necessary, and there is time for a physician to explain the situation in order to obtain the patient's consent, was higher in those cases decided in favor of patients than in those decided in favor of the physicians and/or hospitals (Table 2). Furthermore, patients commonly have to pay the medical and hospital bills for elective treatment, as relatively few of these costs are covered by public health insurance in Japan. A review of elective treatments shows that the majority involve cosmetic surgery or procedures associated with aesthetics. Thus, it is thought that patients may well demand a detailed explanation and doctors might not meet all of the patient's expectations in this respect.

Fifth, regarding the severity of injury of the patient, the death ratio was low with respect to decisions in the patient's favor. Death results did not necessarily influence the judgment of physician liability by the court (Table 2). Previous studies have reported that the rate of decisions in favor of patients was lower when a patient died than when injured temporarily or permanently [20]. The present finding is in line with the previous finding that the severity of injury does not influence a court's decision. The mean age of patients receiving favorable decisions was younger than that of patients who received unfavorable decisions. This might be due to the fact that the prevalence of death or of elective treatment is lower among younger patients. Conversely, it may be inferred that, for elderly patients, obtaining informed consent is considered difficult by virtue of the presence of an underlying disease, resulting in greater chances of unfavorable decisions.

As for the reasons for medical disputes, several studies have revealed an association with the relationship between the doctor and the patient, not the outcome of the management [21]. In the process of medical treatment, the doctor's ability to communicate is considered the fourth skill in the field of medicine, after the abilities to prescribe drugs, treat patients, and perform operations. The present study revealed that court decisions varied according to the doctor's communication skill with patients, which indicates the importance of good communication in the doctor-patient relationship.

### 2. Differences in the doctors' communication skills with patients based on the type of medical facility

This study reveals differences in doctors' communication skills with patients according to the type of medical facility. The first variable is the direct recipient of the doctor's explanation. The doctor's explanation included the family more often in a hospital. This might be due to the fact that the ratios of inpatients and surgical departments are higher in hospitals than in clinics. In Japan, it is assumed that the family plays an important role in the decision-making of the patient; in fact, when physicians hold conversations with the patient or family, there is a decreased probability of a court decision of negligent care [14]. Regarding the doctor's explanatory duty, it is the doctor's decision to choose whom to involve in the conversation. However, we found that the family was included in the hospital group much more often than in the clinic group (Table 3).

The second variable is the number of doctors who explained to the patient and/or family. A single doctor typically provided the explanation to the patient in a clinic, while two or more doctors typically provided the information in a hospital setting, as shown in Table 3. These results are not surprising in that doctors usually work alone in clinics, while several physicians are available to provide information to the patients and their families in hospitals.

A third issue involves the manner of the doctor's explanation, where the ratio of verbal explanation supplemented with other methods is greater than verbal communication alone in the clinic group (Table 3). It has been reported that supplementation of a verbal explanation with documents or supporting materials is vital, but no verifying evidence for this exists. Our analysis also shows that the manner of the doctor's explanation to the patient does not influence the court decision (Table 2). In the hospital, many cases were verbally discussed only with the patient; additional studies would help to clarify this association.

Regarding all the other variables, except for the manner of the doctor's explanation, when the cases in hospital were compared with those in the clinic, the following characteristics were recognized: the ratio of elective or untimely treatment is low, the ratio of the patient's death due to injury is high, the ratio of involvement of the surgical department in patient care is high, the mean age of patients is higher, and the ratio of introducing an expert witness is high. In both the clinic and hospital settings, differences exist with respect to the administration of medical care and patient factors. It is possible that these factors may relate to differences in the manner of doctors' explanations. However, that is beyond the scope of our study.

With respect to the timing of the doctor's explanation, the level of the doctor's explanation to the patient and the level of the doctor's explanation to the family, there was no significant difference between doctors in clinics and doctors in hospitals (Table 3). However, these three communication factors were critical points in court decisions of physician liability (Table 2). One possible explanation for the lack of difference is that physicians in both clinics and hospitals might have acquired such basic communication skills.

Interestingly, a notably higher ratio of court decisions acknowledging physician liability occurred in cases involving clinics than in those involving hospitals (Tables 2 and 3). It has been proposed that medical standards should not vary with the size or type of medical facility. When a doctor's communication skill with patients is assessed in terms of its content and range, the basis of the doctor's explanation should depend on universal medical standards when care is delivered [10]. In other words, no difference in the medical standard and range of a doctor's communication skill with patients, based on the type of medical facility, should exist. However, in recent years it has been argued that the standard of care should be higher in hospitals than in clinics. In 1997, the doctor's communication skill with patients was defined as an important factor of medical treatment in a recent law revision (Medical Law, Article 4, Law No. 205, 1948). Given that a physician's explanation can be regarded as an index of the quality of medical care, more detailed explanations should be the norm in hospitals, as the level of medical care in hospitals is thought to be higher than in clinics. Our findings can be construed to support such an opinion, but further study is necessary to verify this point.

In addition, the following factors might be attributable to our findings: Countermeasures to medical malpractice litigation are more adequate in hospitals than in clinics; and the establishment of a bioethics committee and the presence of a legal adviser are more often expected in hospitals than in clinics. In summary, constant vigilance and discussion concerning medical malpractice issues are undertaken in hospitals, which may contribute to hospitals winning more often when it comes to litigation.

### Limitations of the study and future problems

First, this study does not deal with all the recent court decisions concerning violations of the doctor's duty to explain during the study period in Japan. Thus, a bias may have been introduced because the decisions were published in magazines (i.e., case reports) according to topicality and a new interpretation of the laws. In fact, cases in which decisions favored the patient in general medical malpractice litigation in Japan between 1976 and 1987 constituted only 37.3% [19], whereas our study showed 65%. However, few studies have analyzed these decisions and, thus, few interpretations have been derived in Japan. We believe that our data constitute a useful data source that may provide insight into how physicians' communication skills with patients are related to their liability.

Second, only a few cases were analyzed and, in particular, clinic cases were relatively rare. Further cases must be assessed to clarify differences in doctors' communication skills with patients between clinic and hospital settings.

Despite the abovementioned problems, when the factors of a medical dispute were reviewed, decisions in litigated medical malpractice were regarded as important for the following reasons. First, court decisions in medical litigation cases are the only publicly available information on patient-physician communication in the medico-legal field. Second, the analysis of medical malpractice litigation focusing on the doctor's duty to explain will become increasingly important. Thus far, however, studies using medical malpractice litigation have been based on qualitative analyses. Our study has the following characteristics: the decisions of medical litigation cases were evaluated quantitatively, and our results revealed factors related to the doctor's duty to explain. In summary, we presented a new methodology based on court decisions and reported new findings. Because this is the first report regarding a doctor's duty to explain, future studies are needed to verify the validity of our results.

## Conclusion

The following data were obtained in our study. (1) When a physician's explanation was given before treatment or surgery, and it was relevant and specific, the ratio of decisions of physician liability is lower. (2) Differences in the manner of the doctor's explanation had no influence on the court's decision. (3) The ratio of decisions of physician liability was higher when elective or not urgent treatment was given, and the death of the patient did not influence the decision. (4) Concerning doctors' communication skills with patients in hospitals, the following characteristics were noted: a verbal mode of explanation only, more than two doctors actively participated in the explanation, and the recipients of the discussion included the family. (5) The ratio of decisions of physician liability was higher in clinics than in hospitals.

In conclusion, in Japan, characteristic communication skills with patients related to physician liability were revealed. In comparing such characteristics by the type of medical facility (i.e., hospital and clinic), more cases of physician liability were discovered in clinics than in hospitals, which is unique to our study. Recognition of such negligent actions on the part of doctors is essential to the prevention of medical disputes and to the establishment of patient satisfaction.

## Competing interests

The authors declare that they have no competing interests.

## Authors' contributions

TH gathered data, conducted the statistical analysis, and drafted the manuscript. TT participated in the design of the study and critically revised the manuscript for important intellectual content. AH was responsible for the scientific direction and design of the study and assisted with editing the paper. All authors read and approved the final manuscript.

## Pre-publication history

The pre-publication history for this paper can be accessed here:



## References

[B1] David RA, Rhee M (1998). The impact of language as a barrier to effective health care in an underserved urban Hispanic community. Mt Sinai J Med.

[B2] Zebine E, Razgauskas E, Basys V (2004). Meeting patient's expectations in primary care consultation in Lithuania. Int J Qual Health Care.

[B3] Takayama T, Yamazaki Y, Katsumata N (2002). Relationship between outpatients' anxiety levels in a Japanese oncology setting. Soc Sci Med.

[B4] Localio AR, Lawthers AG, Brenann TA, Laird NM, Hebert LE, Peterson LM, Newhouse JP, Weiler PC, Hiatt HH (1991). Relation between malpractice claims and adverse events due to negligence. N Engl J Med.

[B5] Avery JK (1985). Lawyers tell what turns some patients litigious. Medical Malpractice Review.

[B6] Beckman HB, Markakis KM, Suchman AL, Frankel RM (1994). The doctor-patient relationship and malpractice. Arch Intern Med.

[B7] Hickson GB, Clayton EW, Entman SS, Miller CS, Githens PB, Whetten-Goldstein K, Sloan FA (1994). Obstetricians' prior malpractice experience and patients' satisfaction with care. JAMA.

[B8] Levinson W, Roter DL, Mulooly JP, Dull VT, Frankel RM (1997). Doctor-patient communication. The relationship with malpractice claims among primary care doctors and surgeons. JAMA.

[B9] Nakamura T (1992). *Ishi no setstumei to kanjya no hanndanndouinituite *(Doctor's explanation and patient's decision/consent). Hanrei-Taimuzu.

[B10] Nakamura T (2000). *Ishi no hanndann (sairyo) to kannjya no jikoketteikennnituite *(Doctor's decision and patient's right of self-determination). Hanrei-Taimuzu.

[B11] Matsukura T *Mijyukujimoumaku-shoniyorusitumeijirei to iwayurugendaiigaku no suijyunn *(A case of retinopathy of prematurity about doctor's explanation and standard care of today). Hanrei-Taimuzu.

[B12] (1988). Decision by the Supreme Court, Third Small Bench on January 19, 1988. Hanrei-Taimuzu.

[B13] (1978). Decision by the Fukuoka District Court on February 9, 1978. Hanrei-Taimuzu.

[B14] Hagihara A, Tarumi K (2007). Association between physicians' communicative behaviors and judges' decisions in lawsuits on negligent care. Health Policy.

[B15] (1995). Decision by the Tokyo District Court on July 28, 1995. HanreiJiho.

[B16] Kato S (1995). *Iryokagososyo no gennjyou to tenbo *(The state and view of medical malpractice litigation). Hanrei-Taimuzu.

[B17] Dyer C (2004). Surgeon found liable for not warning of partial paralysis risk. BMJ.

[B18] (1997). Decision by the Takamatsu High Court on February 27, 1996. HanreiJiho.

[B19] Hagihara A, Nishi M, Nobutomo K (2003). The structure of medical malpractice decision-making in Japan. J Law Med.

[B20] Hagihara A, Nishi M, Nobutomo K (2003). Standard of care and liability in medical malpractice litigation in Japan. Health Policy.

[B21] Bhan A, Dave D, Verson SA, Bhan K, Bhargave J, Goodwin H (2005). Risk management strategies following analysis of cataract negligence claims. Eye.

[B22] Mazur DJ (2003). Influence of the law on risk and informed consent. BMJ.

